# The effects of caffeine, nicotine, ethanol, and tetrahydrocannabinol on exercise performance

**DOI:** 10.1186/1743-7075-10-71

**Published:** 2013-12-13

**Authors:** Dominik H Pesta, Siddhartha S Angadi, Martin Burtscher, Christian K Roberts

**Affiliations:** 1 Department of Internal Medicine, Yale University School of Medicine, New Haven, CT, USA; 2 Exercise and Metabolic Disease Research Laboratory, Translational Sciences Section, School of Nursing, University of California, Los Angeles, CA, USA; 3 Department of Sports Science, Medical Section, University Innsbruck, Innsbruck, Austria; 4 Healthy Lifestyles Research Center, School of Nutrition and Health Promotion, Arizona State University, Phoenix, AZ, USA

**Keywords:** Common drugs, Doping, Performance enhancement, Anti-doping

## Abstract

Caffeine, nicotine, ethanol and tetrahydrocannabinol (THC) are among the most prevalent and culturally accepted drugs in western society. For example, in Europe and North America up to 90% of the adult population drinks coffee daily and, although less prevalent, the other drugs are also used extensively by the population. Smoked tobacco, excessive alcohol consumption and marijuana (cannabis) smoking are addictive and exhibit adverse health effects. These drugs are not only common in the general population, but have also made their way into elite sports because of their purported performance-altering potential. Only one of the drugs (i.e., caffeine) has enough scientific evidence indicating an ergogenic effect. There is some preliminary evidence for nicotine as an ergogenic aid, but further study is required; cannabis and alcohol can exhibit ergogenic potential under specific circumstances but are in general believed to be ergolytic for sports performance. These drugs are currently (THC, ethanol) or have been (caffeine) on the prohibited list of the World Anti-Doping Agency or are being monitored (nicotine) due to their potential ergogenic or ergolytic effects. The aim of this brief review is to evaluate the effects of caffeine, nicotine, ethanol and THC by: 1) examining evidence supporting the ergogenic or ergolytic effects; 2) providing an overview of the mechanism(s) of action and physiological effects; and 3) where appropriate, reviewing their impact as performance-altering aids used in recreational and elite sports.

## Introduction

Caffeine, nicotine, ethanol and tetrahydrocannabinol (THC) are widely consumed substances in today’s society [1,2]. Drugs such as smoked tobacco, excessive alcohol or cannabis pose a serious problem for public health and the health care system [3]. Further, these substances are also related as they are or have been on the Prohibited List of the World Anti-Doping Agency (WADA) or are monitored due to their potential performance-altering effect and misuse in sport [4]. It is therefore important to characterize these substances with respect to their physiological mode of action as well as their potential to alter performance, as these substances are not only of significant importance for the general public but also for competitive athletes. The purpose of this brief review is to provide an overview of the evidence supporting the ergogenic or ergolytic effects of the most prevalent drugs in western society.

## Caffeine

### Overview

Apart from water, tea and coffee are among the most popular beverages worldwide. The main pharmacologically active substance in both is the purine alkaloid of the xanthines class, 1,3,7,-trimethylxanthine or caffeine. According to European and North American statistics, ~90% of the adult population consider themselves as daily coffee users with an average daily caffeine consumption of about 200 mg or 2.4 mg/kg/day (about 2 cups of coffee) [5]. It is therefore considered the world’s most widely consumed pharmacologically active substance. Caffeine is both water and fat soluble and is quickly distributed in the body after absorption mainly by the small intestine and the stomach with peaking plasma levels after 15–120 min and a half-life of about 5–6 hours with individual variation [6]. Due to its lipophilic nature, caffeine also crosses the blood–brain barrier [7], and is metabolized by the liver into paraxanthine, theophylline, and theobromine [8].

### Mechanism of action

Caffeine most likely exerts its performance enhancing effect on the human body mainly by five mechanisms:

1. Antagonism of adenosine [9]. Due to its close chemical resemblance of adenosine, caffeine blocks adenosine receptors (mainly A_1_ and A_2A_ receptor subtypes), thereby competitively inhibiting its action [10]. Caffeine can decrease cerebral blood flow [11] as well as antagonize A_1_, A_2A_ and A_2B_ adenosine receptors in blood vessels, thereby reducing adenosine-mediated vasodilatation and consequently decrease myocardial blood flow [12].

2. Increased fatty acid oxidation: increased lipolysis leads to decreased reliance on glycogen use [13]. Caffeine switches the substrate preference from glycogen to fat by increasing hormone sensitive lipase (HSL) activity and inhibition of glycogen phosphorylase activity [14].

3. Caffeine acts as a nonselective competitive inhibitor of the phosphodiesterase enzymes [15]. Phosphodiesterases hydrolyze the phosphodiesterase bond in molecules such as cyclic adenosine monophosphate (cAMP), inhibiting the breakdown of cAMP. cAMP activates lipolysis by activating HSL and is an important molecule in the epinephrine cascade [16]. It further activates protein kinase A, which in turn can phosphorylate a number of enzymes involved in glucose and lipid metabolism [17].

4. Increased post-exercise muscle glycogen accumulation: enhanced recovery by increased rate of glycogen resynthesis following exercise [18]. Battram et al. reported that caffeine ingestion has no effect on glycogen accumulation during recovery in recreationally active individuals [19]. Pedersen et al. recently reported that caffeine (8 mg/kg body weight) co-ingested with carbohydrates (CHO) increases rates of postexercise muscle glycogen accumulation compared with consumption of CHO alone in well-trained athletes after exercise-induced glycogen depletion [20]. Although this issue needs further study in different populations (untrained, trained) and at different time points (during exercise or recovery), caffeine added to postexercise CHO feeding seems to have the potential to improve glycogen resynthesis.

5. Mobilization of intracellular calcium: It has been shown that caffeine can enhance calcium release from the sarcoplasmic reticulum [21] and can also inhibit its reuptake [22]. Via this mechanism, caffeine can enhance contractile force during submaximal contractions in habitual and nonhabitual caffeine consumers [23]. Intracellular calcium favors the activation of endothelial nitric oxide synthase, which increases nitric oxide [24]. Some of the ergogenic effects of caffeine might therefore as well be mediated partly by effecting the neuromuscular system and increasing contractile force [25]. There is, however, still controversy about the translation of results from in vitro studies on muscle preparations to caffeine dose and calcium release in vivo (see below).

As for many pharmacological substances, there is generally more than one potential mechanism explaining the ergogenic effects. This is also true for caffeine which might affect both the central nervous system (CNS) and skeletal muscle [26]. Although questionable, a potential downside is that caffeine also has diuretic properties which can exert ergolytic effects during prolonged endurance events [27]. Caffeine intake at very high doses (>500–600 mg or four to seven cups per day) can cause restlessness, tremor and tachycardia [28].

### Effects on performance

Caffeine reduces fatigue and increases concentration and alertness, and athletes regularly use it as an ergogenic aid [29]. Caffeine-induced increases in performance have been observed in aerobic as well as anaerobic sports (for reviews, see [26,30,31]). Trained athletes seem to benefit from a moderate dose of 5 mg/kg [32], however, even lower doses of caffeine (1.0–2.0 mg/kg) may improve performance [33]. Some groups found significantly improved time trial performance [34] or maximal cycling power [35], most likely related to a greater reliance on fat metabolism and decreased neuromuscular fatigue, respectively. Theophylline, a metabolite of caffeine, seems to be even more effective in doing so [36]. The effect of caffeine on fat oxidation, however, may only be significant during lower exercise intensities and may be blocked at higher intensities [37]. Spriet et al. [13] found that ingestion of a high dose of caffeine before exercise reduced muscle glycogenolysis in the initial 15 min of exercise by increasing free fatty acid (FFA) levels which inhibits glycolysis and spares glycogen for later use. Caffeine’s effect of inhibition of glycogen phosphorylase has also been shown in vitro [14] as well as its effect on increasing HSL activity [38]. The effect of caffeine on adipose triglyceride lipase [39] has not been studied and warrants investigation. Following caffeine administration prior to and after the onset of cycling, Ivy et al. [40] found that plasma free fatty acid levels were increased 30% compared to placebo. This action might be mediated by inhibition of the enzyme phosphodiesterase, thereby yielding higher levels of cAMP, which has been identified as important molecule for glycogen metabolism and lipolysis [41]. Phosphodiesterase inhibition has been observed only at high concentrations [42]. When direct Fick measurements were applied, Graham et al. [43] did not find altered CHO or fat metabolism, at least in the monitored leg. Further research is needed to evaluate the effect of caffeine on lipolysis, especially during higher exercise intensities.

Augmented post-exercise recovery by increased rates of muscle glycogen resynthesis has been observed [18,20]. Pedersen et al. [20] found higher rates of muscle glycogen accumulation after the co-ingestion of caffeine with CHO during recovery in highly trained subjects. This might, at least in part, be mediated by the activation of AMP-activated protein kinase (AMPK) [44] as it is involved in the translocation of glucose transporter 4 (GLUT4) to the plasma membrane. This mechanism enables the cell to take up glucose from the plasma and store it as glycogen. Not only does caffeine impact endurance, it has also been reported to benefit cognitive function and fine motor skills [45]. While the performance enhancing effects of caffeine in moderate-to-highly trained endurance athletes are quite clear and well documented, its effects on anaerobic, high-intensity tasks are less well investigated. Whereas caffeine supplementation did not yield significant performance increases in a Wingate test in untrained subjects [46,47], Mora-Rodriguez et al. [48] report that caffeine ingestion of 3 mg/kg could counter reductions in maximum dynamic strength and muscle power output on the morning (2.5–7.0%) thereby increasing muscle performance to the levels found in the afternoon. Especially with regard to anaerobic performance caffeine’s adenosine receptor blocking effect in the CNS may be important [30]. A possible explanation for the diverging effect of caffeine on anaerobic performance is that caffeine seems to benefit trained athletes who show specific physiological adaptations whereas performance gains in untrained subjects might be lost or masked by a high variability in performance.

It has been shown that coffee, by containing phenolic compounds such as chlorogenic acids, elicits metabolic effects independent of caffeine [49]. These compounds may have the potential to antagonize the physiological responses of caffeine. The question therefore remains whether ingesting the same amount of caffeine via a food source (e.g. energy bar or coffee) is as effective as ingesting isolated caffeine in the form of a tablet. As mentioned above, the performance enhancing effect of caffeine is very clear. Only a few studies, however, have shown a positive effect of coffee on performance. Whereas some studies found enhanced performance after coffee consumption [50-53], others did not [49,54,55].

One of the earlier works by Costill et al. [53] reported increases in time trial performance of competitive cyclists only in the coffee trial group (containing 330 mg caffeine 1 h prior to exercise) but not in the decaffeinated coffee trial.

Graham et al. [49] studied exercise endurance in runners after ingestions of a caffeine (4.45 mg/kg BW) or placebo capsule with water or either decaffeinated coffee, decaffeinated coffee with added caffeine or regular coffee. The authors found that only caffeine significantly improved running time to exhaustion at 75% VO_2max_ but neither did regular coffee or decaffeinated coffee plus caffeine. Based on these results, the authors speculated that some component(s) in coffee possibly interfere with the ergogenic response of caffeine alone.

This is in opposition to Hodgson et al. [52] who looked at time trial performance in trained subjects after administration of caffeine (5 mg caffeine/kg BW), coffee (5 mg caffeine/kg BW), decaffeinated coffee and placebo one hour prior to exercise. The authors report similar significant increases of ~5% in time trial performance in both the caffeine and the coffee supplemented group with no effects in the decaf or placebo group. The authors conclude that coffee consumed 1 h prior to exercise, at a high caffeine dose improved performance to the same extent as caffeine.

One reason for the disparity of the two studies mentioned above might be the different performance tests used. Whereas Graham et al. used a time to exhaustion test which reportedly can exhibit a coefficient of variation as high as ~27% [56], Hodgson et al. used a time trial which have been shown to be more reproducible. It has also been speculated by Hodgson et al. [52] that due to lower statistical power, Graham et al. [49] were not able to detect a difference between caffeine and coffee ingestion on performance. At this point, both coffee and caffeine exhibit a performance enhancing effect. Further research will hopefully extend our understanding on this issue.

Another reason for the widespread use of caffeine within the exercise community might be its small but significant analgesic effect [57], possibly mediated by augmenting plasma endorphin concentrations [58]. It is also established that caffeine reduces the rate of perceived exertion during exercise [59], suggesting that athletes are able to sustain higher intensities but do not perceive this effort to be different from placebo conditions.

Some studies used caffeine-naïve whereas others used caffeine-habituated subjects. There seems to be a higher increase in plasma adrenalin in caffeine-naïves compared to caffeine habituated subjects after caffeine ingestion [60]. However, no differences between habitual caffeine intake and 1500 m running performance [51] or force of contraction [23] could be observed. For both caffeine-naïve as well as caffeine-habituated subjects, moderate to high doses of caffeine are ergogenic during prolonged moderate intensity exercise [61]. Although there is clearly the need to study caffeine habituation further, the differences between users and non-users do not seem to be major.

### WADA status

From 1962 to 1972 and again from 1984 to 2003 caffeine was on the WADA banned list, with a concentration >12 μg/ml in the urine considered as doping. Caffeine has been demonstrated to be ergogenic at doses lower than those doses that result in a urine concentration of 12 μg/ml, and higher doses appear to exhibit no additional performance-enhancing effect [17]. During the second banned period, many athletes tested positive for caffeine. The sanctions ranged from warnings up to 2 year suspensions (maximum penalty, usually only 2–6 months). Since 2004, caffeine has been removed from the prohibited list, however, it is still part of WADAs monitoring program (stimulants - in competition only) in order to monitor the possible potential of misuse in sport. According to WADA, one of the reasons caffeine was removed from the Prohibited List was that many experts believe it to be ubiquitous in beverages and food and that having a threshold might lead to athletes being sanctioned for social or dietary consumption of caffeine [62]. Furthermore, caffeine is metabolized at very different rates in individuals [63] and hence urinary concentrations can vary considerably and do not always correlate to the dose ingested. In addition, caffeine is added to a wide range of popular food products [64] such as coffee, tea, energy drinks and bars, and chocolate.

### Summary

In summary, caffeine, even at physiological doses (3–6 mg/kg), as well as coffee are proven ergogenic aids and as such – in most exercise situations, especially in endurance-type events – clearly work-enhancing [26]. It most likely has a peripheral effect targeting skeletal muscle metabolism as well as a central effect targeting the brain to enhance performance, especially during endurance events (see Table 1). Also for anaerobic tasks, the effect of caffeine on the CNS might be most relevant. Further, post-exercise caffeine intake seems to benefit recovery be increasing rates of glycogen resynthesis.

**Table 1:** Summary of the effects of caffeine on performance

**Caffeine**			
**WADA status: now being monitored (stimulants - in competition only), banned from 1962 to 1972 and again from 1984 to 2003 at urinary caffeine concentrations >12 μg/ml**			
**Acute effect**	**Effect on performance**	**Caffeine dose**	**Reference**
Greater reliance on fat metabolism; increased FFAs; lower respiratory exchange ratio (RER)	Increased time trial performance	6 mg/kg body mass	Mc Naughton et al. [34]
Counteract central fatigue, directed effect on the CNS	3% PMAX increase, increase in voluntary activation, maintenance of MVC	6 mg/kg body mass	Del Coso et al. [35]
No clear mechanism; effect on CNS (greater motor unit recruitment and altered neurotransmitter function) or direct effect on skeletal muscle	Enhanced time trial performance	6 mg/kg caffeine 1 h pre-exercise and ~1.5 mg/kg after 2 h of exercise	Cox et al. [33]
No mechanism proposed	No significant effects observed on performance	1.5 or 3 mg/kg body mass of caffeine 1 h before cycling	Desbrow et al. [65]
Direct effect on skeletal muscle; interaction with ryanodine receptor; potentiated calcium release from the SR	Increase in contraction force at low frequency stimulation (20 Hz)	6 mg/kg 100 min before stimulation	Tarnopolsky et al. [23]
Blunted pain response	Significantly higher reps during leg press set 3 with caffeine, same RPE	6 mg/kg 1 h prior to 10-RM bench and leg press	Green et al. [66]
Glycogen-sparing effect & increased utilization of intramuscular TGs and plasma FFAs with caffeine	Increased cycling time trial performance with caffeine	9 mg/kg body mass 1 h before exercise	Spriet et al. [13]

## Nicotine

### Overview

Nicotine or 3-(1-methyl-2-pyrrolidinyl)pyridine is a naturally occurring alkaloid and one of the most widely used psychostimulants in the world [67]. Cigarettes are the most common source of nicotine. Smoked tobacco contains additional harmful constituents and chemicals, which have detrimental effects on the respiratory system [68]. Due to worldwide smoking restrictions, the tobacco industry has developed a number of smokeless alternatives, often containing much higher nicotine concentrations than regular cigarettes. These represent an alternative for some athletes as they do not pose a risk of adversely affecting the respiratory system.

### Mechanism of action

In general, nicotine has a psychostimulatory effect on the CNS at low doses via enhancing the actions of norepinephrine and dopamine in the brain [69]. At higher doses, however, nicotine enhances the effect of serotonin and opiate activity, exerting a calming and depressing effect [70]. Nicotine-induced stimulation of the sympathetic nervous system leads to increased heart rate and blood pressure [71], cardiac stroke volume and output [72] and coronary blood flow [73]. Although the results are conflicting and some authors report increases in cutaneous blood flow and skin temperature [74], others report a decrease in cutaneous blood flow and subsequent decline in skin temperature associated with nicotine consumption [75,76]. These differences in cutaneous blood flow are possibly related to differences in nicotine administration. Both snus and nicotine gums enable nicotine to diffuse across the mucous membranes and are taken up by the bloodstream or, when inhaled, diffuses across the alveolar membrane of the alveoli, and enters the bloodstream. Although the amount of nicotine inhaled is lower than with conventional cigarettes, the use of electronic cigarettes is becoming more and more popular [77]. However, the amount taken up by smokeless tobacco users tends to be much greater than by smoking. Once in the bloodstream, nicotine is quickly (within seconds) delivered to the brain, where it interacts with neural nicotinic acetylcholine receptors (nAChRs). It is metabolized by the liver cytochrome P450 enzyme system and has a half-life of approximately 2 hours [78]. Upon binding of ACh or its exogenous ligand nicotine, the ion channel is opened and causes an influx of sodium and calcium (Ca^2+^). This local increase in intracellular Ca^2+^ can alter cellular functions [79]. A mechanism termed Ca^2+^-induced Ca^2+^ release can further boost intracellular calcium upon activation of nAChR [80]. In vitro experiments using human neutrophils showed a dose-dependent rise in intracellular Ca^2+^ levels of 700% over baseline at a concentration of 10^-2^ M nicotine [81]. In numerous pathways, Ca^2+^ acts as an intracellular messenger, setting the stage for nAChRs as potent candidates to influence a variety of Ca^2+^-dependent neuronal processes, such as neurotransmitter release, synaptic plasticity or gene transcription.

### Effect on performance

While it is clear that smoking can lead to the development of respiratory, cardiovascular, and skin diseases as well as a number of tobacco-related cancers [82] there are other forms of application such as the use of alternative smokeless tobacco (snus), which is gaining popularity among athletes [83] as it bypasses the respiratory system. Snus and cigarette consumers show similar peak blood nicotine levels after use with a tendency for higher cotinine levels in the former [84].

Nicotine activates the sympathoadrenal system, which leads to increased heart rate, contractility, vasoconstriction and a rise in blood pressure and the level of circulating catecholamines during light exercise [85]. Nicotine also increases muscle blood flow [86] and lipolysis due to enhanced circulating levels of norepinephrine and epinephrine as well as direct action on nicotinic cholinergic receptors in adipose tissue [87]. The effects exerted by nicotine may be beneficial in a wide variety of sports and it is suggested that nicotine is abused by athletes [83]. According to Marclay [88], cumulative exposure to nicotine metabolites were found in 26% - 56% of urine samples that were subjected to screening for tobacco alkaloids. After correcting for exposure to second-hand smoke, 15% of the athletes were considered active nicotine consumers. Among athletes, this is high considering the WHO’s 25% worldwide estimate of smoking prevalence. It can be hypothesized that the metabolites stem mostly from smokeless tobacco due to the adverse effects of conventional cigarettes for athletes, which most severely affects athletes engaging in endurance type sports [89].

Further, a large number of human and animal studies have found nicotine-induced improvements in several aspects of cognitive function, including learning and memory [90], reaction time [91] and fine motor abilities (see Table 2). Studies addressing the question of a direct performance enhancing effect of nicotine are rare but will be summarized here. Sports most affected include ice hockey, skiing, biathlon, bobsleigh, skating, football, basketball, volleyball, rugby, American football, wrestling and gymnastics. These sports seem to gain performance benefits from the stimulating effect of nicotine as evident from the use of other prohibited stimulants according to the Anti-Doping Database [92]. Muendel et al. [93] found a 17% improvement in time to exhaustion after nicotine patch application compared to a placebo without affecting cardiovascular and respiratory parameters or substrate metabolism. In this sense, nicotine seems to exert similar effects as caffeine by delaying the development of central fatigue as impaired central drive is an important factor contributing to fatigue during exercise. To date, no improvement on anaerobic performance (Wingate test) has been reported (see Table 2).

**Table 2:** Summary of the effects of nicotine on performance

**Nicotine**			
**WADA status: in order to detect potential patterns of abuse, nicotine has been placed on WADA’s 2012 monitoring program**			
**Acute effect**	**Effect on performance**	**Nicotine dose**	**Reference**
Likely delayed development of (central) fatigue by nicotine receptor activation and/or dopaminergic pathways; no evidence of altered substrate metabolism or cardiorespiratory effects	17% improvement in time to exhaustion	7 mg nicotine patch per 24 hours	Mundel et al. [93]
No mechanism proposed	No effect on anaerobic performance (Wingate test)	nicotine gum	Meier [94]
Unclear	Improvement in the degree in a real-life motor task, i.e. handwriting (more pronounced in smokers than non-smokers)	2 and (4 mg) nicotine gum	Tucha et al. [95]
No mechanism proposed	No effect on cognitive functioning	2 and 4 mg nicotine gum	Heishman et al. [96]
No mechanism proposed	No effect on speed and accuracy of motor activity among non-smokers (but improvements in smokers)	2 and 4 mg nicotine gum	Hindmarch et al. [97]
Likely by the action of nicotine on cholinergic pathways	Positive effects on fine motor abilities like finger tapping	2 mg intranasal	West et al. [98]

It is important to note that, compared to rest, exercise can lead to increased drug absorption from transdermal nicotine patches, possibly due to exercise-induced increase in peripheral blood flow at the site of the transdermal patch. Lenz et al. [99] report increased plasma nicotine levels and toxicity due to increased drug absorption during physical exercise. To prevent undesirable side effects, health professionals, trainers and coaches should therefore be aware of proper transdermal patch use, particularly while exercising. Athletes are encouraged to inform their physician about their exercise routine before beginning transdermal patch use. Athletes should further be familiar with signs and symptoms of drug toxicity related to the medication contained in the transdermal patch and consult their physician if signs or symptoms arise. Reducing exercise intensity and duration for the first 1–2 weeks until potential side effects are known might also help to minimize toxicity. To reduce increased exercise-induced drug absorption, athletes are encouraged to avoid exercising in extreme environmental and temperature conditions, wear appropriate breathable sports garments and drink plenty of fluids to prevent dehydration [99].

Additionally, although nicotine may have ergogenic potential, it is also highly addictive, reportedly as addictive as heroin and cocaine [100]. Therefore, detrimental effects on motor performance can be altered after a short abstinence duration. Burtscher et al. [101] noted that motor performance declines in heavy smokers after a short period of abstinence appears, this decline being similar to the motor symptoms of Parkinsonism. The abstinence symptoms are ameliorated by cigarette smoking. It is important to consider the concerning addictive potential with following deterioration of motor performance upon abstinence. Interestingly, however, it was noted that moderate and vigorous exercise led to significant reductions of the desire to smoke among abstaining smokers, possibly via reductions in cortisol [102]. A recent meta-analysis showed that exercise has the potential to acutely reduce cigarette cravings and could therefore be a promising strategy to attenuate withdrawal symptoms in smokers [103]. It is also important to mention that the vasoconstriction mediated by nicotine could limit exercise performance in a hot environment. As skin blood flow increases during exercise to transfer heat impaired nicotine-induced skin blood flow may be ergolytic.

A recent meta-analysis conducted by Heishman and colleagues clearly suggests significant effects of nicotine on fine motor abilities, including attention and memory [104]. Participants of the studies included in the meta-analysis were mainly nonsmokers, therefore avoiding confounding of nicotine withdrawal. Considering the importance of cognition in sport, such an optimization of neurobiological function in our view seems to be beneficial for a variety of sports such as sport games or track and field. Finally, nicotine’s effect on increased pain tolerance might be of advantage in a wide variety of sports [105]. More research will hopefully fill the gap to further evaluate nicotine’s effects on exercise performance.

### WADA status

Based on observations of possible extensive smokeless nicotine consumption among certain athletes [106], a recent report by Marclay et al. [88] from the Swiss Laboratory for Doping Analyses in Lausanne caught the interest of the sporting community and the WADA. Thereafter, discussions within WADA took place in the List Committee which is a subcommittee of the Health, Medical and Research Committee. Dr. Arne Ljungqvist, chairman of the Health, Medical and Research Committee, reports that WADA wants to know more about the use of nicotine in sports. Once the prevalence is known, WADA will discuss a potential ban. Ljungqvist also reports that the IOC has already monitored nicotine as far back as 30 years ago in collaboration with the anti-doping laboratory in Cologne, and did not report abuse^a^. Since this time, smokeless tobacco and other nicotine delivery systems that might be appealing to the sporting community have entered the market. As a reaction, WADA has included nicotine, categorized as a stimulant and ‘in-competition only’ in its 2013 monitoring program [4]. For this purpose, WADA [4] reports: “WADA, in consultation with signatories and governments, shall establish a monitoring program regarding substances which are not on the Prohibited List, but which WADA wishes to monitor in order to detect patterns of misuse in sport”.

### Detection

Nicotine use can be detected in urine samples based on its major metabolites (cotinine, trans-3-hydroxycotinine, nicotine-N0-oxide and cotinine-N-oxide) and tobacco alkaloids (anabasine, anatabine and nornicotine) which can be used as an indicator of a person’s exposure to nicotine as suggested by Marclay et al. [88].

### Summary

In summary, nicotine seems to have ergogenic potential. Athletes appear to benefit from activation of the sympathoadrenal system with increased catecholamine release and subsequent increases in muscle blood flow and lipolysis. One component of nicotine action seems to act via a central mechanism (by nicotine receptor activation and/or dopaminergic pathways; see Table 2). There is evidence for the abuse of nicotine by athletes. Although the sale of snus is illegal within the European Union [107], anecdotal observations by coaches and research from Scandinavia shows a high prevalence of snus use among athletes [83,108]. It might therefore be reasonable to assume that smoking cigarettes will not be an issue for athletes. Instead, as there are several nicotine alternatives many of the negative effects of cigarettes can be circumvented.

## Ethanol (alcohol)

### Overview

Alcohol (ethanol; here referred to as alcohol or EtOH) is and has been one the most commonly consumed and abused drugs for a substantial period in human history. Alcohol is a dependence-producing drug which affects a host of organ systems and one that increases the risk of morbidity and mortality from different diseases when abused [109]. Indeed, some authors have suggested that alcohol is harmful similar to drugs such as heroin or cocaine and that excessive alcohol consumption is a serious world-wide health risk [110]. Although the detrimental effects of alcohol on human physiology are well known, even elite athletes consume alcohol. When looking at the effects of alcohol on overall health, it is, however, important to distinguish between chronic, moderate alcohol consumption versus alcohol abuse.

Alcohol consumption and sport have been inextricably linked since ancient times when alcohol was considered the elixir of life [111]. To some extent that may be true, given that a substantial body of epidemiological evidence shows that moderate ingestion of alcohol may reduce the risk of cardiovascular disease [112]. The link between alcohol consumption and mortality is subject to a J-shaped curve i.e. improved longevity with moderate consumption with increasing intake resulting in greater mortality risk [113]. Indeed, dietary guidelines from the American Heart Association recommend moderation of alcohol intake as it has been associated with a lower risk of cardiovascular events [112].

Alcohol use is fairly widespread among the athletic population with 88% of intercollegiate American athletes reporting the use of alcohol [114]. It is also noteworthy that many athletes consume alcohol prior to sports events [115]. However, it is important to note that scientific evidence suggests that the consumption of alcohol has some detrimental effects on exercise performance [109,114]. It is fairly obvious that it is unlikely for competitive athletes to be alcohol abusers and most performance studies have focused on the acute ergolytic effects of EtOH consumption. The chronic studies merely reinforce the point that EtOH is profoundly ergolytic in the long term setting. They also serve to reinforce that chronic EtOH use can be toxic to cardiac and skeletal muscle.

### Mechanism of action

Chronic alcohol abuse has significant detrimental effects on the human cardiac muscle [116] and one of the putative mechanisms via which alcohol may induce cardiac dysfunction is through the induction of increased oxidative stress. Interestingly, exercise training blunted the oxidative damage observed in a rat model of chronic alcohol consumption [117]. The authors suggest that exercise training results in an up-regulation of cardiac antioxidants which may in turn reduce the deleterious effects of chronic alcohol induced oxidative stress.

Acute alcohol use can also have effects on cardiovascular determinants of exercise performance. Lang et al. [118] examined the effects of acute alcohol administration on left ventricular contractility using echocardiography and found that alcohol had a significant depressant effect on the myocardium. Specifically, acute alcohol consumption resulted in a decreased end-systolic pressure-dimension slope and reduced velocity of myocardial fiber shortening [118].

Alcohol has significant effects on skeletal muscle substrate utilization during exercise. Specifically, it has been demonstrated that alcohol consumption decreases glucose and amino acid utilization, which can have adverse effects on energy supply to exercising muscle [119-121]. Ethanol consumption induces hypoglycemia and decreases glucose appearance in plasma by decreasing hepatic gluconeogenesis [122]. Ethanol administration has been shown to worsen skeletal muscle determinants of exercise performance such as muscle capillary density and muscle fiber cross-sectional area [121]. It was shown in vitro that alcohol can inhibit sarcolemmal calcium channel actions thereby potentially impair excitation-contraction coupling and diminishing muscular performance [123].

Muscle capillary density is closely related to the oxidative capacity of skeletal muscle [124]. Greater capillary density also allows for a greater surface area for gas exchange and delivery of metabolic substrates. Long term alcohol consumption is associated with the development of alcoholic myopathy which is characterized by a reduction in skeletal muscle capillarity [125]. Exercise training, however, appears to attenuate these adverse changes [126].

Epidemiological data suggest that moderate alcohol consumption is associated with many salutary changes in blood coagulation and fibrinolysis. However, compelling experimental evidence is lacking and often conflicting [109]. Alcohol can also lead to significant post-exercise perturbations in levels of clotting factors. Moderate post-exercise alcohol consumption resulted in significantly elevated levels of Factor VIII at 5 and 22 hours during the post-exercise milieu [127]. Both circulating catecholamine and vasopressin levels have been implicated in up-regulation of Factor VIII [109]. These factors in turn, have been implicated in the pathogenesis of atherosclerosis in prospective studies [128].

Alcohol and exercise may interact with each other to induce disorders in platelet aggregation which are associated with an elevated risk of cardiovascular and cerebrovascular events. Alcohol intoxication has been shown to be linked to cerebrovascular infarctions in a few case-control studies [129,130]. However, the exact pathological mechanisms of the same are currently unknown.

Alcohol consumption following athletic participation is commonly observed and may be associated with disorders in platelet aggregation. El-sayed et al. [131] demonstrated that alcohol ingestion following exercise was associated with a marked increase in platelet count 1-hour following exercise. Platelet aggregation induced by adenosine diphosphate was found to be reduced when exercise was followed by alcohol consumption [131]. Thus, it appears that ingestion of a moderate quantity of alcohol is associated with delayed recovery of platelet aggregation. It is important to note however, that the performance impact of ethanol consumption mediated post-exercise coagulopathy is unknown.

Acute alcohol consumption is associated with the deterioration of psychomotor skills. A significant difference exists in injury rates between drinkers and non-drinkers in athletic populations. Athletes that consume alcohol at least once a week have almost a 2-fold higher risk of injury compared to non-drinkers and this elevated injury rate holds true for the majority of sports examined [114]. The exact mechanisms that may be responsible for the elevated risk of injury are unknown.

Alcohol may also interfere with the body’s ability to recover from injury. Barnes et al. [132] examined the effects of 1 g/kg body weight alcohol consumption on recovery from eccentric exercise-induced muscle injury. The authors measured peak and average peak isokinetic and isometric torque produced by the quadriceps. Alcohol consumption was associated with significantly greater decreases in torque production (40-44%) 36 hours into recovery. The authors concluded that the consumption of a moderate amount of alcohol after damaging exercise magnified the loss of muscle force production potential.

Finally, there is some evidence to suggest that chronic alcohol consumption may result in a positive energy balance and a potentially obesogenic state. Tremblay et al. [133] reported that chronic alcohol consumption did not result in a reduction in lipid intake and that a dietary regimen that provided a large fraction of energy in the form of alcohol increased the risk for a positive energy balance in a free-living state. It appears that alcohol may have a fat-sparing effect [134] similar to that of carbohydrates and may cause fat gain. Suter et al. [135] suggested that alcohol could result in excess body fat gain especially in the upper body. There is some evidence to suggest that obese individuals may be more susceptible to weight gain and the hyperlipidemic effects of alcohol consumption as compared to lean individuals [136]. This is in contrast to epidemiological studies that report a negative association between alcohol consumption and adiposity [137]. This may be explained by the induction of unregulated futile metabolic cycles that appear to significantly aid in the disposal of excess calories [138]. In general, it appears that the effects of alcohol on body weight are controversial and it is likely that moderate consumption of alcohol that replaces calories from carbohydrates and fat is unlikely to result in weight gain [139].

### Effects on performance

Alcohol has direct and demonstrable effects on athletic performance which may be due to its cardiovascular effects. McNaughton et al. [140] demonstrated significant ergolytic effects in short and middle distance runners. The adverse effects were most prominent in events that were more dependent upon aerobic capacity (i.e. 800 m and 1500 m). However there were no adverse effects observed in the 100 m run. Similarly, Kendrick et al. [141] demonstrated a significant impairment in 60-minute treadmill time trial performance in trained athletes following alcohol ingestion. Heart rate and VO_2_ were significantly elevated in subjects after alcohol ingestion and only 1 out of 4 subjects could complete the run. This may be due, in part, to the significant hypoglycemia that the subjects experienced at the 60 minute time point. Acute alcohol consumption may also result in small but significant reductions in sustained power output. Lecoultre et al. [142] demonstrated that acute ethanol ingestion resulted in a ~4% reduction in average cycling power output during a 60 minute time-trial. However, the detrimental effect of alcohol on aerobic performance seems to be dose dependent with a threshold of 20 mmol/L, upon which the effect becomes significant [143]. Consumption of alcohol 24 hours prior to exercise has also been shown to reduce aerobic performance by 11% [114]. Some studies have failed to show reductions in exercise performance following alcohol consumption [144,145]. However, this may be due to limitations in their experimental design as well as type of exercise used. For instance, the lack of ergolytic effects on exercise performance during bicycle ergometer testing may be due to the fact that using a stationary bicycle ergometer does not place significant motor coordination demands as compared to running.

Alcohol can also impair recovery following exercise. It has been shown that alcohol can impair glycogen resynthesis after prolonged cycling [146]. More importantly, alcohol seems to interfere with protein synthesis, most likely by suppressing the mTOR pathway, which is critical to facilitate repair and hypertrophy following strength training [147].

Although alcohol seems to have an overall ergolytic effect on exercise performance, a well-known athlete reportedly consumed alcohol before a ski downhill competition [148]. This is potentially a dangerous precedent especially since alcohol has significant effects on executive functions such as judgment and decision making while also having significant adverse effects on motor control and coordination. This has to be considered in sports requiring a high level of boldness (downhill skiing, downhill mountain biking) and may have implications for pre-participation testing. In addition, the effects of an alcohol-induced hangover are poorly quantified and as such are relatively unknown and subject to further investigation in humans.

### WADA status

Alcohol is prohibited in-competition only and it is prohibited in the following sports: aeronautic, archery, automobile, karate, motorcycling and powerboating. Until 2010, modern pentathlon was also included in this list.

### Detection

Detection is conducted by breath or blood analysis. The limit (blood tests) eligible for a doping violation is 0.10 g/L.

### Summary

Alcohol is the most commonly consumed drug in athletic communities. The American College of Sports Medicine (ACSM) concludes in its position stand that alcohol consumption adversely affects psychomotor skills and exercise performance while resulting in minimal reductions in maximal oxygen consumption (see also Table 3). The ACSM also recommends that if an athlete must consume alcohol, that they should refrain from alcohol consumption for at least 48 hours prior to competition. Chronic alcohol abuse is associated with significant impairments in cardiac and skeletal muscle. It also slows post-exercise recovery by inhibiting protein synthesis. Thus, alcohol is a uniformly ergolytic agent that has significant detrimental effects on exercise performance and that use of the same during competitive activity should be minimized for athlete safety and so as to maximize athletic performance.

**Table 3:** Summary of the effects of alcohol on performance

**Alcohol**				
**WADA status: alcohol is prohibited by WADA in competition only. The doping violation threshold is 0.10 g/L. As of 2013, alcohol is banned in the following sports: aeronautics (FAI), archery (FITA), automobile (FIA), karate (WKF), motorcycling (FIM) and powerboating (UIM)**				
**Acute effect**	**Chronic effect**	**Effect on performance**	**EtOH Dose**	**Reference**
Reduced left ventricular contractility	Increased left ventricular dimensions and worsened left ventricular dysfunction	Negative effects on cardiac output	1.15 g/kg body weight	Delgado et al. [149]
No mechanism proposed; decreased performance possibly due to reduced myocardial contractility and reduced lung ventilation		Increased 800 m-1500 m run times	0.05 – 0.1 mg/mL blood alcohol concentration	McNaughton et al. [140]
Hypoglycemia at 60-minute time-point		Reduced 60-min, treadmill time-trial performance	25 ml in 150 ml grapefruit juice	Kendrick et al. [141]
No mechanism proposed.		Reductions in sustained power output during cycling times trials.	0.5 ml/kg FFM	Lecoulre et al. [142]

## Tetrahydrocannabinol

### Overview

Cannabis (*Cannabis sativa*) is known for its widespread use worldwide. In total, more than 400 different compounds, distributed by 18 chemical groups, including its most active substance Δ^9^-THC have been detected in different species of cannabis plants. Consumption of THC-containing cannabis products, such as marijuana (herbal cannabis) and hashish (resinous cannabis) are commonly consumed in the form of cigarettes or even in small pipes. In addition, dronabinol, a THC synthetic product, has been approved in many countries to treat medical conditions such as HIV and cancer. The widespread popularity of use of substances derived from cannabis, such as marijuana among young athletes has led to its high detection frequency. In 2012, 7.6 million individuals 12 years of age or older used marijuana on 20 or more days in the past month [150].

### Mechanism of action

The structure of THC was described long before its receptors, CB_1_ and CB_2_ were discovered. CB_1_, which has been primarily found in the CNS, likely explains the central psychotropic effects of marijuana [151]. CB_2_ receptors, on the other hand, are mainly found in sensory tissue mediating an analgesic effect [152]. Anandamide, an endogenous ligand binds to these receptors. Anandemide, however, appears to also signal via other receptors than CB_1_ and CB_2_. Future studies should investigate the effects of exercise on the cannabinoid receptor system and how this is modulated by marijuana use. Numerous cannabimimetics (cannabis receptor agonists) are being developed that have similar pharmacologic effects but limited negative side effects. However, illicit cannabimimetics such as Spice and K2 (synthetic cannabis) with dangerous side effects are on the rise, which is a concerning issue [153].

### Effects on performance

The ergogenic effects of marijuana are questionable, as its performance enhancing effect, if any, has yet to be established. Along these lines, very few studies have tested the effects of marijuana on performance. One of the first studies to evaluate the effects of marijuana smoking on exercise performance was performed by Steadward and Singh [154], who tested the effects of marijuana smoking compared to placebo on several indices of exercise performance. Resting heart rate and both systolic and diastolic blood pressure were significantly elevated at rest after marijuana consumption compared to both control and placebo. Although there was no significant decrease in grip strength, physical work capacity at a heart rate of 170 decreased by 25% compared to placebo. Renaud and Cormier [155] tested subjects 10 min after smoking a marijuana cigarette (containing 1.7% of Δ^9^-THC) of 7 mg/kg of body weight, and noted a slight, but significant decrease in cycle ergometry time to exhaustion. Avakian et al. [156] demonstrated that double-blind administration of marijuana as 7.5 mg of Δ^9^-THC or placebo did not affect blood pressure, ventilation or oxygen uptake during submaximal exercise (15 min at 50% of Vo_2max_), however did increase heart rate and the rate-pressure product at rest and during both exercise and recovery. Tashkin et al. [157] hypothesized that the decrease in exercise performance may be due to its chronotropic effect leading to achievement of maximum heart rate at reduced workloads. Furthermore, detrimental effects on other aspects of performance have also been demonstrated. When subjects were acutely given THC orally (215 µg/kg) acutely, significant deficits in general performance, standing steadiness, reaction time and psychomotor performance were observed over a 5 hour period post-ingestion [158]. Interestingly, in a case report [159], it was documented that in a patient with asthma, a condition characterized by bronchoconstriction, smoking marijuana prior to exercise testing led to bronchodilation and no defect in pulmonary function [160,161]. Thus, if there is any positive effect of marijuana, it likely only indirectly improves performance.

It is conceivable that cannabis may reduce an athlete’s feelings of pre-competition stress and anxiety as a result of the euphoric effect it may produce. Also, because cannabis diminishes alertness and has relaxing and sedative properties, use may be driven by the effects of relaxation, well-being and improved sleep quality. For example, it has been reported that relaxing, pleasure, and improved sleeping were the main motives to use cannabis [162], with the rationale that adequate sleep and being relaxed before competition may lead to optimal performance. However, due to the trade-off of decreased exercise performance, possibly secondary to increases in heart rate and blood pressure, which may alter perceived exertion, marijuana may be considered an ergolytic agent.

### WADA status

The International Olympic Committee included cannabis in the banned substance list beginning in 1989 and since 2004 the World Anti-Doping Agency has prohibited its use for all sports competition [163]. Cannabinoids are substances prohibited in-competition only.

### Detection

Testing for cannabis in the form of marijuana, hashish or other cannabis containing products is performed by urine analysis. The target molecule detected in urine analysis is 11-nor-9-carboxy-Δ^9^-THC, with the limit for a positive test at >15 ng/mL. Detection is determined by gas-chromatography/mass spectrometry, and this threshold distinguishes active users from passive smokers and foods that contain traces of cannabinoids [164-166]. However, it should be pointed out that recently Brenneisen et al. [167] suggested that THC, 11-nor-9-carboxy-Δ^9^-THC and 11-hydroxy-Δ^9^-THC all should be considered as target analytes for cannabis doping.

### Summary

Overall, it appears that cannabis does not have ergogenic potential in sports activities and thus, its inclusion on the banned list is likely a function of its illicitness. As cannabis smoking impairs exercise and psychomotor performance (such as sedative effect, slower reaction times and other psychomotor effects), its ability to serve as an ergogenic aid has been questioned, and is generally considered to be an ergolytic drug (see also Table 4). This is likely due to increase in heart rate and blood pressure, decline of cardiac output and reduced psychomotor activity that have been demonstrated in prior studies.

**Table 4:** Summary of the effect of THC on performance

**TETRAHYDROCANNABINOL**			
**WADA status: the International Olympic Committee included cannabis in the banned substance list beginning in 1989 and since 2004 the World Anti-Doping Agency has prohibited its use for all sports competition [**[163]**]**			
**Acute effect**	**Effect on performance**	**THC dose**	**Reference**
Resting heart rate and both systolic/diastolic blood pressure were significantly elevated at rest	Physical work capacity at a heart rate of 170 decreased by 25% compared to placebo	18.2 mg of Δ^9^-THC	Steadward and Singh [154]
Induced tachycardia at rest	VE, VO_2_ and VCO_2_ were increased above control at ≥50% max effort; Small, but significant reduction in maximal exercise duration; tachycardia up to 80% of maximum effort and during recovery	7 mg/kg marijuana (containing 1.7% Δ^9^-THC)	Renaud and Cormier [155]
Increased heart rate and the rate-pressure product at rest	No effect on blood pressure, ventilation or oxygen uptake during submaximal exercise (15 min at 50% of VO^2max^); increased heart rate and the rate-pressure product during recovery	Smoking 7.5 mg of Δ^9^-THC	Avakian et al. [156]

### Drug interactions

Alcohol acts as a depressant and caffeine as a stimulant of the CNS. If the two substances are consumed together, the psychostimulatory effect of caffeine seems to antagonize the depressive effect of alcohol via incomplete antagonism. Indeed, ingestion of a combination of alcohol and caffeine showed no significant difference from placebo when simple reaction time the amplitude of the evoked potentials was assessed [168]. However, the subject’s feeling of intoxication persisted.

The interaction of caffeine with cannabis seems to be more complex. When caffeine and cannabis were given to rats, memory deficits induced by THC were not attenuated but actually exacerbated [169]. This might have to do with the interaction of caffeine with adenosine A_1_ receptors that also modulate cannabinoid signaling in the hippocampus.

In the presence of nicotine, caffeine exhibits a shorter half-life and faster metabolism [170]. Ethanol on the other hand has been shown to slow caffeine metabolism and increases its half-life [171]. Co-ingestion of caffeine and nicotine exhibit additive effects on cardiovascular parameters such as blood pressure during baseline conditions but less than additive effects during conditions of physical and mental stress and sympathoadrenal stimulation [172].

The section on interactions of drugs is not exhaustive but demonstrates that even for these highly prevalent and socially accepted drugs there are potential interactions. Before drawing conclusions from drug interactions, questions arise such as: is the time to peak psychomotor effect of either drug altered in the presence of the other? Does the magnitude of the peak blood concentration change for either drug in the presence of the other? More studies are needed to validate drug interactions and to address these questions in a performance-related context. In the view of the high prevalence of these drugs this task would provide relevant information.

## Conclusion and perspectives

The physiological effects of the above mentioned substances are well established. However, the ergogenic effect of some of the discussed drugs may be questioned and one has to consider the cohort tested for every specific substance. However, only caffeine has enough strength of evidence to be considered an ergogenic aid. Cannabis and alcohol are ergolytic for sports performance, and nicotine needs confirmation with further research. It is well known that there is intersubject variability in response to every drug [173]. Also, every consumer has to understand potential side effects as well as possible interaction effects, if multiple drugs are consumed. Future research with diverse combinations over a longer duration will be needed to establish the safety and efficacy of drugs and ergogenic dietary aids. Educating societal awareness of the potential dangers from drug intake is of paramount importance. For nicotine, WADA will need to if it wants to move the substance from the monitoring program to the Prohibited List in order to curtail wide-spread use of smokeless tobacco in sports. Apart from the ergogenic effect of nicotine, this would underscore WADA’s effort following the ban of THC to promote a drug-free sport. Whether ergogenic or not, the addictive potential of a drug should always be considered.

## Endnote

^a^Personal communication

## Abbreviations

Ach: Acetylcholine; AMPK: Adenosine monophosphate-activated protein kinase; BW: Body weight; cAMP: Cyclic adenosine monophosphate; CHO: Carbohydrates; CNS: Central nervous system; EtOH: Ethanol; FFA: Free fatty acid; FFM: Fat-free mass; GLUT4: Glucose transporter 4; HSL: Hormone sensitive lipase; IOC: International Olympic Committee; nAChR: nicotinic acetylcholine receptor; THC: Tetrahydrocannabinol; WADA: World Anti-Doping Agency.

## Competing interests

The authors declare that there have no competing interests.

## Authors’ contributions

DHP, SSA, MB and CKR have made substantial contributions to ideas, conception and design of the review and have been involved in drafting the manuscript and revising it critically for important intellectual content. All authors have read and have given final approval of the version to be published.
